# Deliberate self-harm among adolescent psychiatric outpatients in Singapore: prevalence, nature and risk factors

**DOI:** 10.1186/s13034-018-0242-3

**Published:** 2018-07-06

**Authors:** Michelle Siu Min Lauw, Abishek Mathew Abraham, Cheryl Bee Lock Loh

**Affiliations:** Department of Psychological Medicine, Changi General Hospital, 2 Simei Street 3, Singapore, 529889 Singapore

**Keywords:** Deliberate self-harm, Self-harm, Adolescent outpatients, Prevalence, Risk factors

## Abstract

**Background:**

Deliberate self-harm (DSH) is a prominent mental health concern among adolescents. Few studies have examined adolescent DSH in non-Western countries. This study examines the prevalence, types and associated risk factors of DSH in a clinical sample of adolescents in Singapore.

**Methods:**

Using a retrospective review of medical records, demographic and clinical data were obtained from 398 consecutive adolescent psychiatric outpatients (mean age = 17.5 ± 1.4 years, range = 13–19 years) who presented at Changi General Hospital from 2013 to 2015.

**Results:**

23.1% (n = 92) of adolescents engaged in at least one type of DSH. Cutting was the most common type of DSH reported. Females were three times more likely to engage in DSH than males. DSH was positively associated with female gender (odds ratio [OR] 5.03), depressive disorders (OR 2.45), alcohol use (OR 3.49) and forensic history (OR 3.66), but not with smoking behaviour, living arrangement, parental marital status, past abuse or family history of psychiatric illness.

**Conclusion:**

Interventions targeting adolescent DSH should also alleviate depressive symptoms, alcohol use and delinquent behaviours.

## Background

Deliberate self-harm (DSH) refers to the intentional, self-inflicted destruction of bodily tissue without suicidal intent and for reasons not socially or culturally acceptable [1]. DSH generally begins during early-to-mid-adolescence and commonly includes cutting (with a knife or razor), scratching, biting, burning, or hitting oneself [2]. Most adolescents engage in DSH in order to cope with intense negative emotional states such as depression and anxiety [3]. Adolescents may also engage in DSH as an attempt to punish oneself, generate sensations of excitement or stimulation and/or gain attention from others [4]. Although adolescents engage in DSH without lethal intent, it could lead to fatality.

Varying prevalence rates of adolescent DSH have been reported within Western community samples, ranging from 18 to 38% [5, 6], and rates rise up to about 80% among adolescent psychiatric inpatient [7]. Adolescent DSH has been found to occur alongside a range of psychiatric issues such as mood and anxiety disorders, borderline personality traits, alcohol and drug use, conduct problems and an elevated risk of suicide [8–10] as well as psychosocial issues such as severe illness of a parent, parental divorce and poor family structure [4, 8]. Depression, in particular, has been consistently found to be the most common diagnosis among adolescents with DSH [6, 9, 11, 12]. Using a Canadian sample, Asbridge et al. [13] reported that adolescents with elevated depressive symptoms experienced a 40% increase in the total number of DSH acts occurring within the preceding 6 months.

Research on the gender differences in adolescent DSH has been mixed. Some studies have indicated higher prevalence rates (up to threefold) in adolescent females compared to males [6, 12, 14–18], while others failed to report this gender difference [19–21]. Some studies have also noted gender over-representations in the type of adolescent DSH reported, with females reporting more cutting behaviours and males reporting more violence-related behaviours such as hitting, burning or aggressive driving [6, 13, 21]. However, other studies failed to replicate these gender patterns in the type of adolescent DSH reported [9].

Few studies have examined adolescent DSH in non-Western samples. Among adolescents in Japan, the reported annual prevalence of DSH was 1.5% among males and 6.9% among females aged 15–18 years old [22]. In Hong Kong, the overall prevalence was found to be 32.7% among adolescents aged 10–18 years old [23]. Consistent with the gender patterns reported in Western samples, adolescent females were also found to have significantly higher rates of DSH than adolescent males in Singapore [18], Japan [22] and Hong Kong [23, 24]. In Singapore, one published study reported that 23.6% of adolescent patients at a psychiatric outpatient clinic engaged in DSH, and DSH was associated with female gender, mood disorders, adjustment disorders and alcohol use [18]. However, the authors did not examine the different types of DSH behaviours engaged and did not account for variables such as history of abuse and forensic history.

Using a sample of adolescent psychiatric outpatients in Singapore, this follow-up study described the prevalence as well as different types of DSH behaviours engaged. We also investigated gender differences in the prevalence and types of DSH and explored whether gender, primary diagnosis, alcohol use, smoking behaviour, living arrangements, parental marital status, family history of psychiatric illness, history of abuse and forensic history were predictive of adolescent DSH. This study expands existing knowledge about the clinical phenomenology of DSH in Singapore and allows us to monitor trends over time.

## Methods

### Participants and procedures

Data was retrospectively collected from medical records of all new adolescent outpatients referred for psychiatric treatment (ages 13–19) seen at the Psychological Medicine Centre of Changi General Hospital, Singapore, from 2013 to 2015. All data was de-identified and study procedures were approved by the institutional review board at Changi General Hospital.

Each patient’s demographic data (e.g., age, gender, employment, living arrangements and parental marital status) and clinical information (e.g., presence and type of DSH behaviours, primary diagnosis, past abuse, alcohol use, smoking behaviour, family history of psychiatric illness and forensic history) were obtained from routine psychiatric intake interview records. In order to avoid unintentionally assessing treatment effects on DSH behaviours, only data from the intake interview was used. In this study, DSH was defined as the intentional self-inflicted destruction of bodily tissue, without the intention to die and excluding culturally sanctioned procedures. Primary diagnoses were made according to the fourth edition of the Diagnostic and Statistical Manual of Mental Disorders [25].

### Statistical analyses

Data were analyzed using IBM SPSS Statistics Version 19.0. Descriptive statistics were used to describe demographic and clinical variables. Pearson’s Chi square tests were used to analyze the relations between DSH and categorical variables. A value of p < 0.05 was considered statistically significant. Candidate risk factors were screened using univariate logistic regression and variables with a value of p < 0.2 were further analysed using multivariate stepwise logistic regression where variables were entered sequentially into the model, and the model with the best fit controlling for confounding variables was selected.

## Results

The final sample consisted of 398 adolescents (mean age = 17.5 ± 1.4 years) of whom 203 (51%) were males. Majority of the sample were students (n = 316, 79.4%) who lived with both biological parents (n = 299, 75.1%) and had married parents (n = 309, 77.6%). The most common primary diagnosis was depressive disorders (n = 106, 26.6%), followed by adjustment disorders (n = 104, 26.1%) and anxiety disorders (n = 96, 24.1%). 98 adolescents (24.6%) had at least one first degree relative with a psychiatric disorder. About one-fifth of the sample reported current or past history of smoking behaviour (n = 77, 19.3%) and alcohol use (n = 89, 22.4%). Selected demographic and clinical characteristics of the study sample can be found in Table 1. 23.1% (n = 92) of the sample engaged in at least one type of DSH. The most common type of DSH reported was cutting (n = 78), followed by hitting or punching (n = 8), scratching (n = 1) and multiple methods (n = 5; see Table 1).

**Table 1:** Sample demographic and clinical characteristics

Characteristic	N (%)
Age (years old)	
13–16	89 (22.5)
17–19	309 (77.7)
Gender	
Male	203 (51)
Female	195 (49)
Employment status	
Student	316 (79.4)
Employed	60 (15.1)
Not working or studying	22 (5.5)
Living arrangements	
Both biological parents	299 (75.1)
Single biological parent	63 (15.8)
Blended families	17 (4.3)
Other biological relatives	10 (2.5)
Others (e.g., student hostel)	9 (2.3)
Parental marital status	
Married	309 (77.6)
Divorced or unmarried or separated	73 (18.3)
Deceased	16 (4.0)
Main diagnosis	
Depressive disorders	106 (26.6)
Adjustment disorders	104 (26.1)
Anxiety disorders	96 (24.1)
Eating disorders	12 (3.0)
Neurodevelopmental disorders	20 (5.0)
Conduct disorders	9 (2.3)
Psychotic disorders	7 (1.8)
Substance disorders	3 (0.8)
No mental illness	25 (6.3)
Family history of psychiatric illness	
At least one first degree relative	98 (24.6)
Other relatives	43 (10.8)
Current or past history	
Smoking	77 (19.3)
Alcohol	89 (22.4)
Physical or sexual abuse	26 (6.6)
Forensic	18 (4.6)
Type of deliberate self-harm	
Cutting	78 (19.8)
Hitting or punching	8 (2)
Scratching	1 (0.3)
Multiple methods	5 (1.3)

Females were significantly (about threefold) more likely than males to engage in DSH, *𝜒*^2^ (1, *n* = 393) = 28.3, *p* = 0.00, *𝜑* = 0.274. More females (n = 63) compared to males engaged in cutting (n = 15). Those who engaged in hitting or punching were all males (n = 8).

Among adolescents with DSH, 73.9% were female, 44.6% had a depressive disorder as their primary diagnosis, 41.3% had a current or past history of alcohol use, 33.7% had a current or past history of smoking, and 32.6% had a positive family history of psychiatric illness. Most patients with DSH lived with their biological parents (71.7%) and 12% had a past or current history of physical or sexual abuse. Table 2 compares the risk factors of adolescents presenting with and without DSH. DSH was found to be significantly associated with female gender, depressive disorders, alcohol use, smoking and past abuse. These five variables were then further analyzed using stepwise logistic regression, where variables were entered sequentially into the model, and Table 3 illustrates the model with the best fit controlling for confounding variables.

**Table 2:** Risk factors of adolescent psychiatric outpatients with and without DSH

Factor	N (%)		p-value
	With DSH (n = 92)	Without DSH (n = 301)	
Female gender	68 (73.9)	125 (41.5)	< 0.001
Depressive disorders	41 (44.6)	65 (21.6)	< 0.001
Anxiety disorders	15 (16.3)	81 (26.9)	0.053
Adjustment disorders	22 (23.9)	82 (27.2)	0.618
Eating disorders	0 (0)	11 (3.7)	0.134
Neurodevelopmental disorders	1 (1.1)	19 (6.3)	0.085
Psychotic disorders	0 (0)	7 (2.3)	0.305
Conduct disorders	2 (2.2)	6 (2.0)	1.000
Substance disorders	1 (1.1)	1 (0.3)	0.958
Alcohol use	38 (41.3)	50 (16.6)	< 0.001
Smoking	31 (33.7)	44 (14.6)	< 0.001
Family history of psychiatric illness	30 (32.6)	109 (36.2)	0.614
Married parents	68 (73.9)	237 (78.7)	0.407
Living with both biological parents	66 (71.7)	229 (76.1)	0.481
Past abuse	11 (12.0)	15 (5.0)	0.029
Forensic history	8 (8.7)	10 (3.3)	0.061

**Table 3:** Multivariate regression analyses of risk factors associated with DSH

Variable	OR (95% CI)	p-value
Female gender	5.03 (2.772–9.130)	0.000
Depressive disorders	2.45 (1.429–4.187)	0.001
Alcohol use	3.49 (1.933–6.305)	0.000
Forensic history	3.66 (1.164–11.527)	0.026

DSH was significantly associated with female gender (odds ratio [OR] 5.03), depressive disorders (OR 2.45), alcohol use (OR 3.49) and forensic history (OR 3.66), but not with smoking behaviour, living arrangements, parental marital status, family history of psychiatric illness or history of abuse (Table 3).

## Discussion

DSH is harmful to the body and can result in unintentional death. Adolescents who engage in DSH represent a vulnerable and high-risk population. The main goal of this study was to examine the prevalence, nature and associated risk factors of DSH among Singaporean adolescents in an outpatient setting. This will improve our understanding of the population attending psychiatric services for young people, identify at-risk groups for DSH and design targeted interventions for DSH and its associated risk factors.

The prevalence of DSH in this study (23.1%) was similar to an earlier study in Singapore [18]. The consistency in this finding, in addition to the sizeable number of patients involved and the equal gender proportion in the current sample, contributes to the accuracy in estimating prevalence. Given that DSH can sometimes be a secretive act, the consistency in prevalence across time suggests that DSH continues to be a significant feature of adolescents presenting with psychiatric symptoms.

The prevalence of DSH in this study was lower than that generally reported in Western clinical samples [7], although there were similarities in the nature of presentation and some associated factors. This may be unsurprising given the growing impact of the internet on globalization and exposure to Western media, which may facilitate the gradual homogenizing of how mental illnesses and coping behaviours such as DSH manifest worldwide [26].

Consistent with previous research on gender differences in DSH in both Western [6, 12, 14–17] and Asian studies [22–24], female adolescents in this study were about three times more likely to engage in DSH than males. These gender patterns have been suggested to reflect the higher rates of depression and anxiety in females as well as the differential ways in which males and females respond to emotional distress [21]. Males have been known to have a greater risk-taking propensity and tend to engage in more externalizing coping methods, whereas females tend to engage in more internalizing coping methods. Findings also mirror prior research on the associations between DSH and depression, alcohol use and delinquent behaviours [4, 9, 13, 22, 24]. Alcohol use and delinquent behaviours may be associated with disinhibition and recklessness, which may lead to increased DSH. Heavy alcohol use and depression have also been significantly associated with DSH among Hong Kong adolescents [24] and it has been suggested that alcohol use may independently increase DSH, or depression may drive adolescents to self-medicate using alcohol. Although DSH was positively associated with a forensic history in this study, more research on this association is needed as the number in this study was small; only 8 patients with DSH had a forensic history.

DSH was not significantly associated with poor family structure (i.e., not living with both biological parents and not having married parents), positive family history of psychiatric disorder and history of abuse, despite these factors being commonly associated with depression and DSH [4, 8]. However, because the current sample was comprised mostly of adolescents who were in school, not smoking, living with both biological parents who were married, had no positive family history of psychiatric disorder, and had no history of drug use and physical or sexual abuse, it is possible that DSH, as a symptom, may be associated with different risk factors when investigated within different socio-demographic profiles. Singaporean adolescents who engaged in DSH have been found to have higher perceived invalidating home environment, despite being from intact families [27]. This finding suggests the need for further research on how the quality of family relationships, rather than the type of family structure, may be implicated in the development of DSH in Singaporean adolescents. Further, the lack of association between DSH and a positive family history of psychiatric disorder may also be attributed to the lack of accurate report from the adolescent patient. Because of the stigma and common misperceptions towards mental illness in Singapore, many individuals cope by withholding information to avoid discrimination [28]; this may mask the presence of mental illness in some families. Future studies would benefit from the use of more sensitive indicators to investigate DSH and its associations with social and familial stressors and supports.

The positive associations between DSH and female gender, depressive disorders, alcohol use and delinquent behaviours suggest that it may be helpful to refine interventions in order to target these factors. For example, developing group therapy programs tailored for adolescent females, including formal screening tools for depressive disorders in patients presenting with DSH as well as addressing alcohol use and delinquent behaviours which may at times be overlooked during discussions about DSH. Because under-aged alcohol use and delinquent behaviours are often socially influenced, consistent social supports provided via individual and family psychoeducation, case management, psychotherapy groups and a supportive familial environment are important in managing these behaviours.

Several limitations warrant consideration. The cross-sectional design of this study precludes causal conclusions. Future prospective longitudinal studies with data on DSH among Singaporean adolescents are needed. Further, because data from this study were drawn from specialist outpatient clinics, the generalizability of our findings to community or primary care settings may be limited.

## Conclusions

This study adds to our understanding of the phenomenon of DSH behaviours among Singaporean adolescents. Continued research is needed to expand our understanding of DSH and its associated risk factors and to refine interventions to include the most feasible, culturally-sensitive and cost-effective treatment targets on group and individual levels. In particular, clinicians should not only aim to analyze and modify the antecedents or circumstance around which DSH occurs for the adolescent, but also look to minimize associated risk factors such as low mood, alcohol intake and delinquent behaviours. Furthermore, DSH has been increasingly described within the scientific literature since the 1970s, it would be interesting to see whether and how changes in the affected population and associated factors over time may impact on the meanings, motivations and manifestation of DSH presenting in adolescents.

## Abbreviations

DSH: deliberate self-harm
OR: odds ratio
